# The Consequences of a Foul Mouth

**Published:** 1900-12-15

**Authors:** 


### HOSPITAL CLINICS AND MEDICAL PROGRESS.

### THE CONSEQUENCES OF A FOUL MOUTH.

SOME time ago Dr. William Hunter[1] put forward a somewhat startling hypothesis in regard to the origin of that strange condition commonly spoken of as pernicious anæmia. This disease he regarded as due in the first instance to septic conditions within the mouth, and the cases by which he supported his contention were such as to convince many, although, doubtless, many more remained entirely sceptical as to there being any causal relation between the conditions there described. Dr. Hunter[1] has now, however, gone back a stage, perhaps on the principle of "reculer pour mieux sauter," and has grouped together a considerable string of morbid states which can be more directly traced to oral sepsis, and when, on the one hand, we consider what we now know as to the effects produced upon many tissues by constantly feeding them with toxic matters, and when, on the other hand, we remember the position of the mouth at the head of the stream which passes constantly through the digestive tract and in a secondary manner through the blood vessels and the lymph canals, we cannot but agree with Dr. Hunter as to the seriousness of the consequences which are likely to follow the implantation of septic infection in such a locality.

Starting with the mouth itself, Dr. Hunter describes the local effects of oral sepsis showing that it may lead to dental caries, gingivitis of all degrees, periostitis, pyorrhœa alveolaris, and deposition of tartar; that in the jaws it may produce periostitis, osteitis, alveolar abscess, and diseases of the bone, and that in the surrounding parts it may lead to pharyngitis, otitis, glandular enlargements, cellulitis, and in some cases thrombosis of veins, ethmoidal suppurration, and meningitis by direct extension. All these conditions are septic in their nature, and are produced by pus-forming organisms. Of all pus-forming organisms, however, those engaged in septic conditions of bone are by far the most virulent, and it seems as if that mixed form of infection which is met with in dental caries were the most provocative of the results described. It must be remembered also that the mere presence of such pus-forming organisms in the mouth is not in itself to be regarded as a disease. Septic inflammation is a question of dose and of resistance. It is when the dose is excessive, as when many teeth are involved, or when the resistance of the mucosa is diminished, that infective processes are set up and disease is produced. Such diseases may not merely take the form of a local stomatitis, but may extend downwards so as to produce a definite condition of septic gastritis; it may also set up as remote effects the whole series of infections which we associate with the septicæmia; and lastly it may produce various toxic effects. The conditions of most immediate interest are however the septic conditions of stomach and the toxic conditions of system that at result from oral sepsis.

The continuous swallowing of mouthfuls of pus organisms is not tolerated indefinitely by the stomach. The number of such organisms that enter that organ is, in some cases, very large. Fortunately, most of them are destroyed by the gastric juice, but not all. A very considerable proportion of such organisms are to be found in the stomach contents, for it is only when the acidity of the gastric juice is considerable—e.g., an hour or two after food—that it exercises any direct bactericidal action. Thus in long-standing dental disease we have precisely those conditions which are most likely to produce direct infection of the stomach: on the one hand a diminished resistance, i.e., a diminished acidity due to chronic indigestion and catarrh; and on the other hand an increased dose, i.e., increased supply of pus-organisms from the necrotic teeth, reaching the stomach, not only during digestion, but in the intervals between meals when the free HCl is at a minimum.

Everyone will admit, as the result of daily experience, that in connection with diseased teeth, disturbances due to abnormal fermentive processes are apt to arise in the stomach. What, however, Dr. Hunter holds is that the effect of constantly swallowing these organisms is not limited to a mere fermentation of food products, but that actual infection of the mucosa with pathogenic microbes may occur. Thus a septic catarrh is set up which is continuously sustained by the influx of septic organisms; and if this chronic inflammatory process be kept up long enough it leads to the usual result of a glandular catarrh, viz., glandular atrophy with increase of the interstitial tissue around. We must then endeavour to rid our minds of the old ideas so long current in regard to the relation between bad teeth and indigestion. In the minds of some this relation seems a mechanical one. Carious teeth cause imperfect mastication; increased and unnecessary work is thrown upon the stomach, and this in turn leads to various forms of indigestion; while in the minds of others the relation is inverted, for they regard the bad teeth as a sign of ill-health, the outcome rather than the cause of the stomach disturbance. In both of these views there is no doubt some truth; but Dr. Hunter points out that by far the most important link between dental disease and indigestion arises from the fact that septic teeth are a continued cause of septic poisoning and of septic gastric infection. Take the cases which one is constantly meeting with of gastric catarrh associated with dental and oral sepsis. "The ashy-grey look and general languor which such patients, in one's experience, characteristically present are really manifestations of long-continued septic absorption; the local symptoms of clamminess of the mouth, distaste for food, coated tongue, and bad taste in the mouth, which one simply looks upon as manifestations of gastric catarrh, are really the result of oral sepsis; while the nausea, indigestion, and gastric discomfort are the result of 'septic' gastric catarrh produced by direct infection of the stomach with the pus-organisms." Coming to the illustrative cases themselves, Dr. Hunter shows not only the frequency with which such cases may be met with, but also the extraordinary way in which the most remarkable conditions of oral sepsis are apt to be overlooked, even while the patient is being all the time treated for their effects; and he also shows, what it is important to remember, that the gastric catarrh does not cease as soon as the condition of oral sepsis from which it has arisen is removed, and that we need not therefore be surprised at its persistence when, as is often the case, infection from the mouth proceeds continuously for years. Leaving now the question of local septic inflammation, whether in the mouth, the stomach, or the intestinal canal, we have to consider the results of the constant absorption

B

of the products of such inflammation into the system—its toxic results.

These toxic effects are, we are told, extremely common and no less commonly overlooked. The commonest are a "dirty, ashy-grey look and general languor, irritability, feelings of intense depression." Then there may be fever, of obscure character, really septic; septic rashes may occur; purpuric hæmorrhages and bleeding from the gums may arise; and there may be a condition to which Dr. Hunter now draws attention for the first time—namely, nervous effects denoting deep-seated changes in the nervous system, which may be included under the title "Toxic Neuritis." Now, no one can doubt the great importance of Dr. Hunter's observations, which, as it seems to us, may give the key to the pathology of many an obscure case, and may place in the hands of the practitioner a clearly defined and scientific means of relieving many a patient on whom doctor after doctor has played up and down the whole gamut of stomach remedies without avail. What Dr. Hunter especially insists upon is that not only the patient, but the doctor and the very dentist who deals with the teeth themselves too often overlook the septic condition of the mouth, which is the root of the mischief. The physician called in to treat gastric disorders is content to ascribe them to errors in food or drink, or to habits of eating, and treats them for years with stomachic medicines, while he overlooks the stomatitis which is at the root of the matter. The surgeon who is punctilious to a degree in regard to all the details of his operations; whose whole life, it may be said, is passed in excluding and in combating septic infection—even he will perform the most complicated and severe operations on the stomach or intestinal canal without the slightest regard to the presence of septic teeth, septic roots, septic conditions of the gums, and buccal mucous membrane. The dentist, who does so much for his patient in these days of conservative dentistry and high professional dental skill, and who on the strength of his experience can reproach the physician for his neglect of unhealthy conditions of the mouth—even he "will skilfully gold-cap a tooth, or put on a gold bridge, or supply a patient with tooth plates: the gold cap to cover a diseased and blackened tooth; the bridge to form a compact and inaccessible pocket for the growth of pus-organisms between itself and the gum; the tooth-plate to be worn for years without any cleaning other than scrubbing, and as often as not covering foul septic necrotic stumps, or so ill-fitting that rather than be troubled with their removal the patient allows them to grow into the gums." Evidently we are all in the same boat and are all miserable sinners. Even the pathologist is not exempt from this general anathema. In regard to treatment it must be remembered that this is not a mere matter of ordering a mouth-wash, but involves the search for and the direct treatment of each lesion by strong antiseptic solutions applied directly; the removal of all diseased stumps and roots, in particular those lying underneath any tooth-plate; and the avoiding of any dental apparatus liable to become septic which cannot be removed.

[1] Lancet, Vol. 1, 1900, pp. 221, 296, and 371. [2] Practitioner, December.

